# Supplementary material for: The strength of association between psychological factors and clinical outcome in tendinopathy: A systematic review
Source: PLoS One. 2020 Nov 30;15(11):e0242568. doi: 10.1371/journal.pone.0242568 (PMC7703922; doi:10.1371/journal.pone.0242568)
Supplement: Attachment — Submitted filename: PONE-D-20-23274_reviewer-1.pdf [file pone.0242568.s002.pdf]

PLOS ONE

The strength of association between psychological factors and clinical outcome in tendinopathy: a systematic review

--Manuscript Draft--

**Manuscript Number:**	PONE-D-20-23274
**Article Type:**	Research Article
**Full Title:**	The strength of association between psychological factors and clinical outcome in tendinopathy: a systematic review
**Short Title:**	The strength of association between psychological factors and clinical outcome in tendinopathy
**Corresponding Author:**	Sean Mc Auliffe<br>Qatar University<br>Doha, QATAR
**Keywords:**	Tendinopathy; psychological; Association
**Abstract:**	Objective Tendinopathy is often a disabling, and persistent musculoskeletal disorder. Psychological factors appear to play a role in the perpetuation of symptoms and influence recovery in musculoskeletal pain. To date, the impact of psychological factors on clinical outcome in tendinopathy remains unclear. Therefore, the purpose of this systematic review was to investigate the strength of association between psychological and clinical outcome in tendinopathy.<br>Methods A systematic review of the literature and qualitative synthesis of published trials was conducted. Electronic searches of ovid MEDLINE, ovid EMBASE, PsychINFO, CINAHL and Cochrane Library was undertaken from their inception to June 2020. Eligibility criteria included RCT's and studies of observational design incorporating measurements of psychological factors and clinical outcome in participants with tendinopathy. Risk of Bias was measured using a modified version of the Newcastle Ottawa Scale. High or low certainty evidence was examined using the GRADE criteria.<br>Results Ten studies of observational design (6-cross sectional and 4 prospective studies), involving a sample of 719 participants with tendinopathy were included. Risk of bias for the included studies ranged from 12/21 to 21/21. Cross-sectional studies of very low certainty evidence revealed significant weak to moderate strength of association (r = 0.24 to 0.53) between psychological factors and clinical outcomes. Prospective baseline data of very low certainty evidence showed weak strength of association between psychological factors and clinical outcome. However, prospective studies were inconsistent in showing a predictive relationship between baseline psychological factors on long-term outcome.<br>Conclusion Cross sectional studies report similar strengths of association between psychological factors and clinical outcomes in tendinopathy to those found in other musculoskeletal conditions. However, the overall body of the evidence after applying the GRADE criteria was very low certainty evidence, due to risk of bias, imprecision and indirectness found across included studies. Future, high quality longitudinal cohort studies are required to investigate the predictive value of baseline psychological factors on long-term clinical outcome.
**Order of Authors:**	Sean Mc Auliffe
	Carl Stubbs
	Terrence Haines
	Adrian Mallows
	Kieran O'Sullivan
	Peter Malliaras
**Additional Information:**	
**Question**	**Response**

Powered by Editorial Manager® and ProduXion Manager® from Aries Systems Corporation

**Financial Disclosure**<br><br>Enter a financial disclosure statement that describes the sources of funding for the work included in this submission. Review the submission guidelines for detailed requirements. View published research articles from *PLOS ONE* for specific examples.<br><br>This statement is required for submission and **will appear in the published article** if the submission is accepted. Please make sure it is accurate.<br><br>**Unfunded studies**<br>Enter: *The author(s) received no specific funding for this work.*<br><br>**Funded studies**<br>Enter a statement with the following details:<br>• Initials of the authors who received each award<br>• Grant numbers awarded to each author<br>• The full name of each funder<br>• URL of each funder website<br>• Did the sponsors or funders play any role in the study design, data collection and analysis, decision to publish, or preparation of the manuscript?<br>• **NO** - Include this sentence at the end of your statement: *The funders had no role in study design, data collection and analysis, decision to publish, or preparation of the manuscript.*<br>• **YES** - Specify the role(s) played.<br><br>* typeset	The author(s) received no specific funding for this work
**Competing Interests**<br><br>Use the instructions below to enter a competing interest statement for this submission. On behalf of all authors, disclose any competing interests that could be perceived to bias this work—acknowledging all financial support and any other relevant financial or non-financial competing interests.<br><br>This statement **will appear in the published article** if the submission is accepted. Please make sure it is	The authors have declared that no competing interests exist.

accurate. View published research articles from *PLOS ONE* for specific examples.<br><br>**NO authors have competing interests**<br><br>Enter: *The authors have declared that no competing interests exist.*<br><br>**Authors with competing interests**<br><br>Enter competing interest details beginning with this statement:<br><br>*I have read the journal's policy and the authors of this manuscript have the following competing interests: [insert competing interests here]*<br><br>* typeset	
**Ethics Statement**<br><br>Enter an ethics statement for this submission. This statement is required if the study involved:<br><br>• Human participants<br>• Human specimens or tissue<br>• Vertebrate animals or cephalopods<br>• Vertebrate embryos or tissues<br>• Field research<br><br>Write "N/A" if the submission does not require an ethics statement.<br><br>General guidance is provided below. Consult the submission guidelines for detailed instructions. **Make sure that all information entered here is included in the Methods section of the manuscript.**	N/A

**Format for specific study types**<br><br>**Human Subject Research (involving human participants and/or tissue)**<br>• Give the name of the institutional review board or ethics committee that approved the study<br>• Include the approval number and/or a statement indicating approval of this research<br>• Indicate the form of consent obtained (written/oral) or the reason that consent was not obtained (e.g. the data were analyzed anonymously)<br><br>**Animal Research (involving vertebrate animals, embryos or tissues)**<br>• Provide the name of the Institutional Animal Care and Use Committee (IACUC) or other relevant ethics board that reviewed the study protocol, and indicate whether they approved this research or granted a formal waiver of ethical approval<br>• Include an approval number if one was obtained<br>• If the study involved *non-human primates*, add *additional details* about animal welfare and steps taken to ameliorate suffering<br>• If anesthesia, euthanasia, or any kind of animal sacrifice is part of the study, include briefly which substances and/or methods were applied<br><br>**Field Research**<br><br>Include the following details if this study involves the collection of plant, animal, or other materials from a natural setting:<br>• Field permit number<br>• Name of the institution or relevant body that granted permission	
**Data Availability**<br><br>Authors are required to make all data underlying the findings described fully available, without restriction, and from the time of publication. PLOS allows rare exceptions to address legal and ethical concerns. See the PLOS Data Policy and FAQ for detailed information.	Yes - all data are fully available without restriction

Powered by Editorial Manager® and ProduXion Manager® from Aries Systems Corporation

A Data Availability Statement describing where the data can be found is required at submission. Your answers to this question constitute the Data Availability Statement and **will be published in the article**, if accepted.<br><br>**Important:** Stating 'data available on request from the author' is not sufficient. If your data are only available upon request, select 'No' for the first question and explain your exceptional situation in the text box.<br><br>Do the authors confirm that all data underlying the findings described in their manuscript are fully available without restriction?	
**Describe where the data may be found in full sentences. If you are copying our sample text, replace any instances of XXX with the appropriate details.**<br><br>• If the data are **held or will be held in a public repository**, include URLs, accession numbers or DOIs. If this information will only be available after acceptance, indicate this by ticking the box below. For example: *All XXX files are available from the XXX database (accession number(s) XXX, XXX.).*<br>• If the data are all contained **within the manuscript and/or Supporting Information files**, enter the following: *All relevant data are within the manuscript and its Supporting Information files.*<br>• If neither of these applies but you are able to provide **details of access elsewhere**, with or without limitations, please do so. For example:<br><br>*Data cannot be shared publicly because of [XXX]. Data are available from the XXX Institutional Data Access / Ethics Committee (contact via XXX) for researchers who meet the criteria for access to confidential data.*<br><br>*The data underlying the results presented in the study are available from (include the name of the third party*	All relevant data are within the manuscript and its Supporting Information files

and contact information or URL).<br>• This text is appropriate if the data are owned by a third party and authors do not have permission to share the data.<br><br>* typeset	
Additional data availability information:	

Powered by Editorial Manager® and ProduXion Manager® from Aries Systems Corporation

Manuscript

Click here to access/download;Manuscript;Association between psychological factors in tendinopathy.docx

The strength of association between psychological factors and clinical outcome in tendinopathy: a systematic review

Carl Stubbs[1¶], Sean Mc Auliffe[2¶*], Adrian Mallows[3¶], Kieran O'sullivan[4¶] Terence Haines[5&], Peter Malliaras[6¶]

¶ These authors contributed equally to this work

& These authors also contributed equally to this work

* sean@qu.edu.qa

1 Sunshine Coast Hospital Health Service, Queensland, Australia; Monash University. **2** Department of Physical Therapy and Rehabilitation Sciences, College of health Sciences, Qatar University, Doha, Qatar. **3** School of Health and Human Sciences, University of Essex, Colchester, UK. **4** Department of Allied Health, University of Limerick, Limerick, Ireland. **5** Department of Physiotherapy, School of Primary and Allied Health, Faculty of Medicine Nursing and Health Science, Building B, Peninsula Campus, Monash University, Victoria, Australia 3199.**6** School of Primary and Allied Health Care, Faculty of Medicine Nursing and Health Science, Building G, Peninsula Campus, Monash University, Victoria, Australia 3199.

Abstract

Objective Tendinopathy is often a disabling, and persistent musculoskeletal disorder. Psychological factors appear to play a role in the perpetuation of symptoms and influence recovery in musculoskeletal pain. To date, the impact of psychological factors on clinical outcome in tendinopathy remains unclear. Therefore, the purpose of this systematic review was to investigate the strength of association between psychological and clinical outcome in tendinopathy.

Methods A systematic review of the literature and qualitative synthesis of published trials was conducted. Electronic searches of ovid MEDLINE, ovid EMBASE, PsychINFO, CINAHL and Cochrane Library was undertaken from their inception to June 2020. Eligibility criteria included RCT's and studies of observational design incorporating measurements of psychological factors and clinical outcome in participants with tendinopathy. Risk of Bias was measured using a modified version of the Newcastle Ottawa Scale. High or low certainty evidence was examined using the GRADE criteria.

Results Ten studies of observational design (6-cross sectional and 4 prospective studies), involving a sample of 719 participants with tendinopathy were included. Risk of bias for the included studies ranged from 12/21 to 21/21. Cross-sectional studies of very low certainty evidence revealed significant weak to moderate strength of association (r = 0.24 to 0.53) between psychological factors and clinical outcomes. Prospective baseline data of very low certainty evidence showed weak strength of association between psychological factors and clinical outcome. However, prospective studies were inconsistent in showing a predictive relationship between baseline psychological factors on long-term outcome.

Conclusion Cross sectional studies report similar strengths of association between psychological factors and clinical outcomes in tendinopathy to those found in other musculoskeletal conditions. However, the overall body of the evidence after applying the GRADE criteria was very low certainty evidence, due to risk of bias, imprecision and

indirectness found across included studies. Future, high quality longitudinal cohort studies are required to investigate the predictive value of baseline psychological factors on long-term clinical outcome.

Introduction

Tendinopathy, previously referred to as tendinitis or tendinosis, is a common musculoskeletal condition characterised clinically by pain reported around the affected tendon with loading [1]. Tendinopathy affects both athletic and non-athlectic populations alike. For example, Achilles tendinopathy is reported in up to 50% of runners before the age of 45 years [2]. In general practice, Albers et al. [3] reported lower extremity tendinopathy prevalence rates of 11.8 per 1000 person-years, whilst prevalence rates for upper limb tendinopathies have been estimated between 1.3% to 21.0% [4, 5, 6].

Recommended care for tendinopathy includes exercise interventions involving progressive exercise interventions such as concentric and/or eccentric strengthening [7, 8]. Success rates reported using such exercise programmes in Achilles tendinopathy have been shown to vary between 56% and 100%. [9, 10] whilst moderate response rates of 41% have been reported for eccentric exercise in people with lateral elbow tendinopathy [11]. Given the often persistent and multifactorial nature of tendinopathy, similar to to other MSK disorders, treatments in tendinopathy may also need to address the multiple factors that contribute to pain, dysfunction and disability experienced.

It has been suggested that psychological factors such as fear of reinjury, pain catastrophising, external locus of control and low self efficacy may negatively impact on clinical outcomes in common musculoskeletal disorders [12-15]. However, to date, the contribution of

psychological factors to the pain, dysfunction and disability experienced in tendinopathy and the benefits of focussing upon theses factors as treatment targets remain uncertain. Recent qualitative studies have outlined the negative psychological impact of persistent Achilles tendinopathy, rotator cuff tendinopathy and greater trochanteric pain syndrome [16-19]. Likewise, a recent cross-sectional study reported greater levels of psychological distress and poorer quality of life among patients with more severe gluteal tendinopathy [20].

Previous systematic reviews [21, 22] concluded that there was a conflicting evidence-base for the association of psychological factors in people with tendinopathy and did not provide detail regarding the strength of association. If psychological factors were shown to be strongly associated with disease severity and if trials of management approaches that focus on these factors show them to be beneficial, it would provide stronger justification for targeting these factors in real-life management. To address this, we aimed to identify and synthesise available evidence investigating the strength of association between psychological factors and clinical outcomes in tendinopathy.

Methods

Protocol details were registered in the international prospective register of systematic reviews (PROSPERO - registration number CRD42019139757) and reported in accordance with the Preferred Reporting Items for Systematic Reviews and Meta-Analysis (PRISMA) [23].

Eligibility criteria

Studies investigating a relationship between (i) psychological domains and (ii) clinical measures of pain and/or disability/function in tendinopathy were deemed suitable for inclusion. Randomised controlled trials (RCTs), quasi-experimental studies, cross-sectional uncontrolled trials, case-control, case series and cohort studies were eligible. Reviews, case studies, editorials, and studies with between group analysis were excluded.

Participants

Studies which included adult participants labelled as having a tendinopathy of any duration, affecting the upper (rotator cuff, lateral elbow tendinopathy) or lower limb (plantar heel pain, Achilles tendinopathy, patellar tendinopathy, gluteal trochanteric pain syndrome), were included. In keeping with recent recommendations tendinopathy diagnosis could be based on clinical history and assessment, excluding other potential diagnosis, with or without imaging [1].

Outcome measures

Primary outcomes

Self-reported psychological instruments commonly reported in musculoskeletal literature measuring the following psychological factors were deemed suitable for inclusion in the review [24, 25].

- **Emotional factors** including, but not limited to, depression, distress, anxiety, hypervigilance/somatisation, stress and anger.
- **Cognitive factors** including, but not limited to, maladaptive beliefs, fear, kinesiophobia, catastrophising, negative pain beliefs, self-efficacy.

Secondary outcomes:

An a-priori decision was made to include the following clinical parameters relating to pain and disability as secondary outcomes: objective physical function, pain, self-reported disability, and any adverse effects.

- **Objective physical function:** Including but not limited to strength (isometric, isotonic, endurance), heel raise test, hop, balance and jumping tests.
- **Self-reported disability:** The data were extracted from the function scale highest on the following tendinopathy function scale hierarchy when trialists reported data for more than one scale.
    - Victorian Institute of Sports Assessment (VISA-P VISA-A, VISA-G).
    - Any other tendon specific disability scale (SPADI, GROC)
- **Pain** – Including, but not limited to, pain severity, overall pain, daily pain, pain with activity - including mean pain or change in mean overall pain measured via visual analogue scale (VAS), numerical rating scale (NRS) or categorical scale.
- **Adverse effects**: Number of participants experiencing an adverse effect (as defined by the trial authors)

Timing of outcome assessment

Primary and secondary outcomes were extracted and categorised into the following:

- Up to 4-weeks (short-term)
- > 4-weeks and up to 3-months (medium-term)
- > 3-months (long-term)

Data sources and search

A systematic search of the following databases was performed by the lead author (CS): ovid MEDLINE, PsychINFO, Cochrane Library, ovid EMBASE and CINAHL plus. The search was originally performed in May 2019 and updated in June 2020. A sensitive search strategy using relevant search terms was developed from Medical Subject Headings (MeSH) and keywords were used (see Table 1). The complete search strategy for each database is shown in Appendix 1. Relevant grey literature was also searched via OpenGrey and ongoing trials via the National Institute of Health (clinicaltrials.gov). The electronic search was supplemented by hand searching of references lists from included articles.

Table 1. Keywords used in the search strategy

Main search terms
1. Tendinitis or tendinopathy or epicondylitis or tennis elbow or shoulder impingement syndrome or rotator cuff injury or jumper's knee (MeSH)
2. Fear or depression or anxiety or catastrophizing or coping behaviour or avoidance behaviour or emotion or self-efficacy (MeSH)
3. 1 and 2 combined

Selection of studies

Database screening was conducted by one author (CS) who independently screened titles and abstracts for potentially eligible trials based on predetermined criteria. Any potentially eligible studies along with studies whose abstract did not provide enough information, were retrieved for full-text review and independently assessed by two authors (CS & AM) to determine eligibility. In cases of disagreement an agreement was made by consensus, and if required a third author was consulted (PM).

Data extraction

Two authors (CS & PM) independently extracted data from each study to a standardised form. Disagreements were resolved through consensus, or through a third reviewer (SM) if required.

The following data were extracted from each study where available (see Table 2):

- Study characteristics (first author, year of publication, type of trial [e.g. case control], country, source of funding, sample size, trial registration [If registration number is reported]).
- Patient characteristics (mean age, mean duration of symptoms, sex, educational status, physical activity status, work status).
- Outcomes (psychological measures, function, disability, pain intensity, adverse events – as detailed above)

Table 2. Characteristics of Included Studies

First Author/type of study	No of participants	Mean age (years)	Clinical presentation	Duration of symptoms	BMI	Psychological factors outcome measures	Pain outcome measures	Disability Outcome measures	Physical Functional Outcome measures	Data Collection (follow up)	Statistical Results
Akyol et al.[37] 2013<br><br>Cross sectional study	45	52.69±11.99 (SD)	Subacromial Impingement syndrome	11.47±13.69 (SD) months	28.93 ± 4.91(SD)	Depression (BDI)<br><br>QoL & Mental Health (SF-36)	(VAS 0-10) SPADI – pain VAS subscale	(SPADI)	SRS (IR) and (ER) velocity of 60°/sec and 180°/sec	T1 Baseline	Depression negative association with SRS 60º/s ERPT (r= - 0.477, p= 0.001). Depression negative association with SRS 180°/s IRPT (r= -0.332, p= 0.026). Depression negative association with SRS 180°/s ERPT (r= -0.526, p=0.001). Mental Health sub scale of SF-36 negative association with SRS 60°/s ERPT (r= -0.345, p=0.020). Mental Health negative association with SRS 180°/s ERPT (r= - 0.361, p=0.015). Non-significant associations between Mental Health and SRS 60°/s & 180°/s IRPT. Depression was not significantly associated with SRS 60°/s IRPT.
Coombes et al[43]. 2015<br><br>Cohort prognostic study	41	49.9 ± 7.4 (SD)	Lateral elbow tendinopathy	25.7 ± 28.7 (SD) Weeks	-	Kinesiophobia – (TSK -17), 17-68, (where 0 is best) Depression/anxiety (HADS) 14 items rated on 4-point scales with a total score ranging from 0 to 42, Anxiety (0-21 with 0 being best); Depressive symptoms (0-21 with 0 being best).	Pain intensity (PRTEE) 5-items to assess pain scored on a 10-point likert scale (0-best)	Functional Disability (PRTEE) – 10-items on 10-point likert scale (0 is best).	-	(T1) at baseline (T2) at 2-months (T3) at 1-year	Psychological factors depression,anxiety or kinesiophobia(TSK, HADS) did not significantly influence either pain (PRTEE), disability (PRTEE) at 2 or 12-months

First Author/type of study	No of participants	Mean age (years)	Clinical presentation	Duration of symptoms	BMI	Psychological factors outcome measures	Pain outcome measures	Disability Outcome measures	Physical Functional Outcome measures	Data Collection (follow up)	Statistical Results
											HADS, per point at 52-weeks (β = -0.210; p = 0.087)
Cotchett et al.[40] 2015 Cross sectional study	84	56.07 ± 12.19 (SD)	Plantar heel pain	13.6 ± 12.2 (SD)Months	29.25 Range 20.9-44.30	Depression/anxiety/stress (DASS)- 0 to 21 for each subscale, where 0 is best and 42 is worst	FHSQ - Sub-SCALE, 0 is worst foot pain - 100 is no foot pain	-	FHSQ - (0 is best foot function - 100 is worst foot function)	(T1) baseline	Depression showed significantly negative association with foot function (β= -0.28, p=0.009).<br>Stress showed significantly negative association with foot function (β = -0.29, p=0.006)<br>Depression had a negative association with foot function in females (β= -0.53; p<0.001).<br>Depression was a significant predictor of foot pain in a model with females (β=−0.41; p= 0.013)<br>Stress was a significant predictor of foot pain in females in this model (β=−0.36; p=0.024) but not in males<br>The association between stress and foot function was significant for females (β=−0.50; p=0.001) but not significant for males (β=0.01; p=0.929)<br>Anxiety was not a significant predictor of foot pain when added to a model with age, sex and BMI (β=−0.04; p=0.744); or when

First Author/type of study	No of participants	Mean age (years)	Clinical presentation	Duration of symptoms	BMI	Psychological factors outcome measures	Pain outcome measures	Disability Outcome measures	Physical Functional Outcome measures	Data Collection (follow up)	Statistical Results
											included in a model with stress (β=−0.04; p=0.783) or depression (β =−0.02; p= 0.892).
Cotchett et al.[41] 2017<br><br>Cross sectional study	36	47.3 ± 13.2 (SD)	Plantar heel pain	10.3 ± 12.8 (SD) Months	24.6 ± 12.0 (SD)	Kinesiophobia (TSK-17) score 17-68, (where 0 is best)<br>Pain catastrophising (PCS) score 0-52 (0 being best)	Pain intensity (FHSQ, SCALE, 0 is worst foot pain - 100 is no foot pain);	-	FHSQ - (0 is best foot function - 100 is worst foot function) First step pain (0-100 VAS, where 0 is best)	(T1) baseline	Univariate analysis Significant association between kinesiophobia and foot function (r = -0.47, p=0.005) but not pain (r = 0.01, p= 0.96). Significant association between catastrophising and foot function (r=-0.62, p=0.000) and pain (r=0.37, p=0.025).<br>Multivariate analysis Kinesiophobia had a negative association with foot function (R^2 change 0.21, F(1,31) = 8.73, p = 0.006)<br>Catastrophising had a negative association with foot function R^2 of 0.39 f(1,31) =21.142, p < 0.001)<br>Catastrophising explained 39% of the variability in foot function (β = −0.65, P < 0.001).<br>Catastrophising and first step pain – R^2 0f 0.18, F(1,31) = 7.918, P = 0.008 catastrophising accounted for 18% of the variability in first step pain (β = 0.44, P = 0.008).

First Author/type of study	No of participants	Mean age (years)	Clinical presentation	Duration of symptoms	BMI	Psychological factors outcome measures	Pain outcome measures	Disability Outcome measures	Physical Functional Outcome measures	Data Collection (follow up)	Statistical Results
Engebretson et al.[36] 2010<br><br>Cross-sectional study	200	49.8 ± 10.9 (SD)	Subacromial shoulder pain	Between 3-months and >24 months		Emotional distress-anxiety and depression (HSCL-25) Consists of 25 items: Part I of the HSCL-25 has 10 items for anxiety symptoms; Part II has 15 items for depression symptoms. Scale rated 1-4 Overall score 25-100 with (25 being best)	Pain intensity during rest and activity (9-point Likert scale 1-9 with 1 being best)<br><br>SPADI – Pain subscale	SPADI - 0-100 where 0 is best.	-	(T1) baseline	Univariate regression Distress HSCL-25 significant association with SPADI total B = 13.9 (7.6 to 20.2) p<0.001<br>HSCL-25 significant association with SPADI pain subscale B=13.6 (7.0 to 20.3) p<0.001<br>HSCL-25 significant association SPADI disability subscale B=14.9 (7.8 to 21.9) p<0.001<br>Distress HSCL-25 significant association with SPADI total on multivariate regression B = 9.6 (3.0 to 16.2) P<.001<br>Distress HSCL-25 significant association on multivariate regression with SPADI pain subscale B = 10.1 (3.0 to 17.2) P=0.006<br>Distress HSCL-25 significant association with SPADI on multivariate regression on disability subscale B= 10.4 (3.3 to 17.5) P < 0.001
Ferrer- Pena et al.[44] 2019<br><br>Cross - sectional study	51	49.14 ± 8.693 (SD)	Greater trochanteric pain syndrome	20.06 ± 28.658 months	27.29 ± 5.62 (SD)	PCS score 0-52 (0 being best)<br>TSK, 11 where 11 is best. Student t-test for independent samples was used to compare the outcomes between	Pain intensity (VAS 0-10 where 0 is best)	Spanish version of WOMAC - 24 items divided into 3 subscales -	Dynamic balance YBT. YBT composite score is calculated by summing the three reach directions (Forward, posteromedial	(T1) - baseline	Anterior YBT movement, negative associations between PCS and YBT-F p < 0.05. PCS subscale of helplessness revealed

First Author/type of study	No of participants	Mean age (years)	Clinical presentation	Duration of symptoms	BMI	Psychological factors outcome measures	Pain outcome measures	Disability Outcome measures	Physical Functional Outcome measures	Data Collection (follow up)	Statistical Results
						subjects who had lower levels of kinesiophobia with those with higher levels of kinesiophobia. No cut offs given.<br>CPSS- 22 item questionnaire each item 10-point Likert scale anchored on the ends by 10 = very' uncertain and 100 = very certain (best = higher score)		scored on a scale of 0-4,. Pain 0-20 Stiffness 0-8 Physical function - 0-68 (0=best)	posterolateral measured in cms) and normalizing to lower extremity length.<br>YBT Forward established as criterion variable.		negative association - p<0.05<br>Positive associations were found in the pain subscale of the CPSS for anterior 0.432**, postero-medial 0.313*, and postero-lateral movements 0.309, other correlations for CPSS subscales not significant.<br>Kinesiophobia was not significantly associated with three movements of YBT.<br>Criterion variable (YBT-F) was predicted by VAS measures, the WOMAC total score, and the TSK-11 total score in multiple linear regression analysis. TSK-(B=0.267, p=0.02) Adjusted R^2 explaining 43.8% of variance
Harutaichun et al.[42] 2019<br><br>Prospective cohort study	71	21.54 ± 1.14 (SD)	Plantar Fascitis	Between 2 and 10-weeks	23.37 ± 3.81(SD)	DASS (0 to 21 for each subscale, where 0 is best and 42 is worst)	Pain intensity (VAS) - No details regarding type of VAS (0-10 where 0-is best)	-	Ankle dorsiflexion angle subject in prone with knee extension measured with styandar goniometer<br>Ankle plantarflexion strength - single leg heel raise - elevate heel every 2s in time with metronome. (norm 25 raises)	(T1) Baseline	Depression and pain intensity positive association (r=0.27, p = 0.03)<br>Anxiety and pain intensity positive association (r = 0.38, p = 0.01)<br>Stress and pain intensity positive association (r= 0.27, p = 0.03)<br>Multiple linear regression analysis

First Author/type of study	No of participants	Mean age (years)	Clinical presentation	Duration of symptoms	BMI	Psychological factors outcome measures	Pain outcome measures	Disability Outcome measures	Physical Functional Outcome measures	Data Collection (follow up)	Statistical Results
									Lateral step-down test from 15-cm step height. Score based on 5-criteria with scores range from 0-6 (where 0 = best)		revealed participants with PF with reported higher levels of pain scored higher with anxiety – From the regression model there was a 0.13(cm) increase in pain intensity for each point of anxiety score. (*B* 0.41, p < 0.01)
Kromer et al.[38] 2014<br><br>Cross-sectional longitudinal analysis	90	51.8 (11.2) (SD)	Sub acromial shoulder pain	38 (114) Median (IRQ)	-	FABQ, a 16-item questionnaire consists of 2-subscales (FABQ-PA) only one used. 4 items of scale are scored on a 7-point Likert scale (0-strongly disagree, 6-strongly agree (0 = best)<br>PCS score 0-52 (0 being best)	Pain intensity (11-point VNRS)<br>SPADI pain sub scale	SPADI contains 5 items assessing pain and 8 items assessing shoulder function. Only the SPADI-F in study. Each item is scored on a 100-mm visual analogue scale (0 = best)	-	(T1) Baseline<br>(T2) 3-months	Baseline catastrophising and pain intensity positive association (r= 0.318, p<0.01).<br>Baseline Fear avoidance and pain -nonsignificant association -0.061 p>0.05<br>Baseline fear avoidance and disability positive association (r= 0.237, p<0.05)<br>Baseline catastrophising and disability positive association (r= 0.369, p<0.01)<br>Fear avoidance beliefs (PA) positive association with baseline functional subscale (SPADI-F) on hierarchical regression analysis (*B* = 0.287, p = 0.006)<br>Catastrophising non-significant association with baseline functional subscale (SPADI-F) on

First Author/type of study	No of participants	Mean age (years)	Clinical presentation	Duration of symptoms	BMI	Psychological factors outcome measures	Pain outcome measures	Disability Outcome measures	Physical Functional Outcome measures	Data Collection (follow up)	Statistical Results
											hierarchical regression analysis<br>PCS and FABQ-PA non-significant association on SPADI-F at 3-months.
Maestroni et al.[39] 2020<br><br>Exploratory cross-sectional cohort study	67	21.54 ± 1.14 (SD)	Rotator cuff related shoulder pain	8-months (2-108) min, max.	(IQR) 23.5 (22.0-25.4)	Depression and anxiety. Two valid single questions were taken from the Outcome Evaluation Questionnaire. 11-point scale with the endpoints 0 to 10 (where 0 is best)<br>Fear avoidance - Single question asked from Pain and Impairment Relationship Scale 0 – "completely disagree" or 10 – "completely agree" (where 0 is best).	Measured using NRS, where 0 = no pain and 10 = pain as bad as it could be.	Disability was measured UEFI-20. 20 activities and the patient give a score to each one of them<br>The highest score is 80 and the lowest is 0. A lower score indicates higher disability.	Maximal isometric strength of the shoulder internal and external rotator muscles. The isometric testing was performed in prone position with the arm supported in 90° of abduction and neutral rotation. Used electronic hand-held dynamometer	T1 - Baseline	Significant positive association depression and pain intensity- regression analysis co-efficient 0.19 CI-(0.03 to 0.36) p=0.020<br>Significant positive association anxiety and pain intensity Co-efficient 0.29 CI- (0.14 to 0.43) p<0.001<br>Significant negative association depression and disability Co-efficient −1.08 CI- (−1.84 to −0.32) p=0.006<br>Significant negative association anxiety and disability – Co-efficient 1.08 CI-(−1.81 to −0.34) p=0.005<br>Non -significant associations between fear avoidance beliefs and pain and disability<br>Psychological factors not included in multivariable analysis.
Silbernagel et al.[45] 2010<br><br>Case series	34	51.6 ± 8.2 (SD)	Achilles Tendinopathy	4.8 6 ± 0.2 years (SD)	-	TSK-SV-17 items, and the total score varies between 17 and 68. A high score indicates high degree of kinesiophobia.	-	VISA-A-S score ranges from 0 to 100; a lower score indicates	Jump tests - drop countermovement jump and hopping.<br>Strength test - Concentric heel rise; eccentric-	(T1) 0-months<br>(T2) 3-months<br>(T3) 6-months	Kinesiophobia and heel-rise recovery significant negative correlation (r = ---590, p=0.05)<br>TSK-SV(17) was no different between

First Author/type of study	No of participants	Mean age (years)	Clinical presentation	Duration of symptoms	BMI	Psychological factors outcome measures	Pain outcome measures	Disability Outcome measures	Physical Functional Outcome measures	Data Collection (follow up)	Statistical Results
								more symptoms and greater limitation of physical activity	concentric heel rise (peak power in watts)<br>Endurance Test - standing heel-rise with 10% of bodyweight added. (performed in joules).	(T4) 1-year<br>(T5) 5-years	people who were asymptomatic, continued symptoms, or new symptoms at 5 years

BMI, Body Mass Index; **BDI**, Becks Depression Inventory; **CPSS**, Chronic Pain Self-Efficacy Scale; **DASS**, Depression Anxiety Stress Scales; **ER**, External Rotation; **ERPT**, External Rotation Peak Torque; **FABQ**, the Fear-Avoidance Beliefs Questionnaire; **FABQ-PA**, Fear Avoidance Beliefs Questionnaire-Physical Activity; **FHSQ**, the Foot Health Status Questionnaire; **HADS**, the Hospital Anxiety and Depression Scale;; **HSCL**, the Hopkins Symptoms Checklist; **IR**, Internal Rotation **IRPT**, Internal Rotation Peak Torque; **NRS**, Number Rating Scale; **OMPSQ-SF**, Orebro Musculoskeletal Pain Screening Questionnaire-Short Form; **PRTEE**, Patient Rated Tennis Elbow Evaluation; **PCS**, the Pain Catastrophising Scale; **SF-36**, the Short Form (36) Health Survey; **SSP**, Somatic trait anxiety Swedish University Scales of Personality; **SPADI**, the Shoulder Pain and Disability Index; **SPADI-F**, the Shoulder Pain and Disability Index; **SRS**, Shoulder Rotation Strength; **TSK**, the Tampa Scale for Kinesiophobia; **TSK-SV**, Swedish version of the Tampa Scale for Kinesiophobia; **UEFI**, Upper Extremity Functional Index; **VAS**, Visual Analogue Scale-Function; **VISA-A-S**, the Swedish Version of the Victorian Institute of Sports Assessment Achilles Questionnaire; **VNRS**, Verbal Numerical Rating Scale; **WOMAC**, Western Ontario McMaster Universities Osteoarthritis Index; **Y-BT**, Y-Balance Test

Risk of bias and overall certainty

Two reviewers (CS & AM) independently assessed the risk of bias of each individual study using the Newcastle-Ottawa Scale (NOS) see Table 3. The NOS is a review tool for evaluating risk of bias in non-randomised studies [26, 27]. An adapted version of the scale was selected to evaluate all studies because many were single cohort cross-sectional observational studies [28, 29]. The tool consists of four domains of risk of bias assessment; (i) selection bias; (ii) performance bias; (iii) detection bias and; (iv) information bias. Seven items assess the four domains and each item is scored from zero (high risk) to three (low risk), with a total maximum score of 21 points. We then rated the overall risk of bias in each study as high (0-6), moderate (7-13), or low (14-21) in line with previous studies [29, 30].

Table 3. Risk of Bias (Newcastle Ottawa Scale- Modified version)

	Selection bias	Performance Bias		Detection Bias		Information bias			
**First Author**	**A**	**B**	**C**	**D**	**E**	**F**	**G**	**TOTAL**	**Level of bias**
Akyol et al.[37] 2013	2	0	0	3	3	3	3	14/21	Low
Coombes et al.[43] 2015	3	1	1	3	3	3	3	17/21	Low
Cotchett et al.[40] 2015	3	3	2	3	3	3	0	16/21	Low
Cotchett et al.[41] 2017	3	1	2	3	3	3	0	14/21	Low
Engebretsen et al.[36] 2010	2	3	2	3	3	1	1	15/21	Low
Ferrer-Pena et al.[44] 2019	2	3	0	2	3	3	3	14/21	Low
Harutaichun et al.[42] 2019	3	3	3	3	3	3	3	21/21	Low
Kromer et al.[38] 2014	3	3	2	3	3	3	0	16/21	Low
Maestroni et al.[39] 2020	2	3	3	3	3	0	3	14/21	Low
Silbernagel et al.[45] 2010	3	0	0	3	0	3	3	12/21	Moderate

A. Is the source population representative? **B**. Is the sample size adequate and is there sufficient power? **C**. Did the study adjust for confounders? **D**. Did the study use appropriate statistics to measure outcome of interest? **E.** Is there little missing data and was it handled appropriately? **F**. Are the methods of outcome measurements Explicitly stated and it is appropriate? **G**. Is there an objective assessment of outcomes?

The overall certainty of the evidence was assessed by two teams of researchers (CS with either PM or SM) using the Grading of Recommendations Assessment, Development and Evaluation (GRADE) approach (see Table 4). The GRADE system classifies the certainty of evidence into four levels; high (further research is very unlikely to change our confidence); moderate (further research is likely to have an impact on our confidence); low (further research is very likely to have an important impact on our confidence); and very low (any estimate effect is very uncertain) [31].

Table 4. Summary of findings and quality of evidence assessment (GRADE)

Outcome by tendon	No. of studies	Types of studies	No. of participants	Risk of bias	Inconsistency	Indirectness	Imprecision	Publication bias	Level of certainty
Rotator Cuff related Shoulder pain	4-studies	3 x cross-sectional<br><br>1 x cross-sectional longitudinal	402	Serious (-1)	No	No	No	Undetected	Very low-level evidence
Plantar Heel Pain	3-studies	2 x cross-sectional<br><br>1 x cohort prognostic study	191	Serious (-1)	No	No	Serious (-1)	Undetected	Very low-level evidence
Lateral Elbow Tendinopathy	1- study	1 x cohort prognostic study	41	Serious (-1)	No	No	No	Undetected	Very low-level evidence
Greater Trochanteric Pain Syndrome	1-study	Cross sectional	51	Serious (-1)	No	Serious (-1)	Serious (-1)	Undetected	Very low-level evidence
Achilles Tendon	1-study	Case Series	34	Serious (-1)	No	No	Serious (-1)	Undetected	Very low-level evidence

The certainty of evidence is based upon five criteria (risk of bias, imprecision, inconsistency, indirectness and publication bias). As all eligible studies were observational designs, all studies started with a 'low quality' rating. Grading upwards was warranted if: 1) a large magnitude of effect existed; 2) there was a dose-response gradient, and 3) all plausible confounders and other biases increased our confidence of estimated effect [32].

Data Synthesis

Due to the heterogeneity of the included studies regarding body site, psychological domains measured, interventions and outcome measures we were unable to pool data from individual studies to perform a meta-analysis. Qualitative synthesis was undertaken with studies grouped specific to each tendon site. Where possible, confidence intervals were calculated from Pearson's r effect sizes and unstandardised and standardised β values from individual studies. Pearson's r values between 0 - 0.2 very weak correlation; 0.2- 0.5 weak correlation; 0.5 – 0.7 moderate correlation; and >0.7 strong correlation [33]. We calculated CI's for Pearson's r correlation coefficients using R Core team) [34, 35].

Results

Study selection

4,152 studies were identified through searching electronic databases. We screened 3,562 titles and abstracts after removing duplicates and identified 44 for full-text review. A total of 10 observational studies (6-cross sectional and 4 prospective studies [3-cohort and 1-case series]) satisfied our eligibility criteria and were included in the final review. Figure 1 illustrates the selection process. We did not find any RCT's or quasi-experimental interventional studies in tendinopathy that had an intervention focussed on psychological factors.

Characteristics of included studies

A total of 719 participants (49.1% females) with tendinopathy were included in the eligible studies. The mean age of participants across the included studies was 48 years of age with a mean duration of symptoms of 14.5 months. Four studies investigated rotator cuff related shoulder pain (subacromial impingement syndrome, subacromial shoulder pain, rotator cuff related shoulder pain) [36-39], three investigated plantar heel pain [40-42], one investigated lateral elbow tendinopathy [43], one investigated greater trochanteric pain syndrome [44] and the remaining study by Silbernagel et al. [45], investigated Achilles tendinopathy (see Table 2).

Outcome measures

The outcome measures utilised across the 10 included studies are detailed in Table 2. For psychological factors, 9-separate psychological outcome measures were used to assess a variety of emotional (4 separate outcome measures) and cognitive (6 separate outcome measures) domains. The most common emotional factors measured were depression (4 studies, 4 separate outcome measures) and anxiety (3 studies, 3 separate outcome measures). The most common cognitive factor measured was kinesiophobia (4 studies, 3 outcome measures), see Table 2.

For the secondary outcomes, 10 studies included 6 separate outcome measures for pain with pain intensity using the visual analogue scale being the most frequent (3 studies). Objective physical function was measured in 7 studies and utilising 10 separate outcome measures, including isometric and isokinetic strength, balance, jumping and hopping. Finally, 7 studies used 5 separate outcome measures for self-reported disability, all of which were region-specific. No studies reported adverse effects which is not surprising given none were trials.

Risk of bias

The overall risk of bias for 9/10 studies was low whilst one study was considered to be at moderate risk of bias. Scores ranged from 12/21 to 21/21 (see Table 3). The most common sources of bias were; not adjusting for confounders, (81.8%), inadequate sample size and statistical power (54.5%) and a lack of objective outcome assessment (45.6%).

Overall certainty of evidence

Due to heterogeneity regarding tendon sites, psychological variables and outcome measures investigated, we were unable to apply the GRADE criteria to measure the certainty of evidence for each individual patient outcome. Consequently, the GRADE criteria was used to establish the certainty of association as it was intended and this was also equivalent to each individual study. Overall, the GRADE criteria demonstrated very low levels of certainty (see Table 4). Limitations mostly related to risk of bias (80%), imprecision (40%) and indirectness (10%). Observational studies included in our review had a greater potential for risk of bias due to a lack of randomisation which increases the possibility of confounding, and selection bias [46]. When considering imprecision, we rated down the evidence quality in four of our studies due to the lack of reporting of 95% confidence intervals.

Associations between psychological factors and clinical outcomes

The specific associations investigated for each study are detailed in Table 2. Some representative examples are provided for each tendinopathy/tendon region below.

Rotator cuff tendinopathy

There was very low certainty evidence from one study of prospective design [38] supporting a weak positive baseline association between catastrophizing and pain ($r = 0.32$, $p<0.01$) and disability ($r = 0.37$, $p<0.01$). Fear avoidance beliefs measured at baseline appeared to be

significantly associated with baseline disability (r = 0.24, p<0.05) but not significant with disability change scores after 3-months.

There was very low certainty evidence from three cross-sectional studies supporting a weak positive association between baseline psychological factors (including depression, anxiety and emotional distress) and pain [36, 39], disability [36, 39] and a negative association with physical function [37]. Thirty four percent (10/29) of the associations investigated were not significant, (refer to Table 2) with some associations differing between clinical outcomes. For example, Kromer et al. [38] showed fear avoidance behaviour was weakly associated with baseline disability (r = 0.237 (95% CI=0.03 to 0.42) p<0.05) but not pain intensity. Furthermore, preliminary cross-sectional associations found between psychological factors and disability were not always evident when confounding variables were considered using multivariate regression models [36] or when measured at long-term follow up [38].

Plantar heel pain

Very low-level certainty evidence from a prospective cohort study found a weak positive association between pain intensity and both depression (r = 0.25 (95% CI=0.02 to 0.45) p<0.03 and stress (r = 0.27 (95% CI=0.04 to 0.47 p<0.03) among men with plantar heel pain [42]. Two cross sectional studies demonstrated a weak to moderate association between psychological factors (kinesiophobia, catastrophizing, depression and stress) and foot function and pain [40, 41]. Forty seven percent (16/34) of associations investigated were not significant (refer to Table 2). Once again, the associations between clinical and psychological factors varied between studies. For example, cross sectional analysis revealed anxiety was not significantly associated with foot pain [40], whereas, a recent prospective cohort study showed anxiety to be the strongest predictor of pain intensity in people with plantar heel pain (β= -0.41, p=0.01) [42].

Lateral elbow tendinopathy

Very low certainty evidence from one cohort prognostic study suggested psychological factors (depression and kinesiophobia) were not significantly associated with either pain or disability in patients with lateral elbow tendinopathy at 2 or 12-months [43].

Greater Trochanteric pain syndrome

Very low certainty evidence from one cross-sectional study investigated the relationship of psychological factors (including kinesiophobia, self-efficacy and catastrophizing) and objective physical function (dynamic balance) [44]. Those with higher catastrophising (helplessness component) were negatively associated with poor performance in components of the Y-balance Test (r= -0.304 (95% CI = -0.53 to - 0.03) $p<0.05$) whilst greater self-efficacy (pain component) revealed a weak positive association with the anterior component of the Y-Balance Test (r= 0.432 (95% CI = 0.18 to 0.62) $p<0.01$). Multiple linear regression analysis revealed greater kinesiophobia, along with higher pain and poorer WOMAC total score, contributed to a poorer score on the anterior component of the Y-Balance Test.

Achilles tendinopathy

There was very low certainty of evidence from a 5-year prospective case series by Silbernagel et al.[45] showing a significant moderate correlation between the level of kinesiophobia (fear of movement) and performance in a repeated standard heel raise functional test (r= -0.590 (95% CI=-0.80 to -0.2 $p<0.005$).

Discussion

This systematic review investigated the relationship between psychological variables and clinical outcomes in tendinopathy. Ten observational studies were included, including six cross-sectional and four prospective studies. Among the cross-sectional studies, there was very low certainty evidence for an association between emotional (e.g. stress, depression) and cognitive (e.g. kinesiophobia, fear-avoidance) issues and greater self-reported pain and disability as well as impaired physical function in people with tendinopathy. In addition, there was very low certatinty evidence for an association between higher levels of self-efficacy and lower levels of pain intensity. In the context of our review the GRADE evaluation suggests we have very low certainty in the associations identified in this review due to factors such as risk of bias, imprecision and indirectness, hence we have very little confidence in the effect estimate [31]. Four prospective studies revealed a conflicting association between psychological factors and change in clinical outcomes. This is a key limitation of this literature, propsective studies are required to explore cause and effect relationships [47].

The strength of association in our cross-sectional data (r = 0.24 to 0.53) is comparable to other reviews among people with other musculoskeletal conditions [30, 48, 49]. In a review of 118 cross-sectional studies in low back pain, Kroska et al.[48] reported very weak-to-moderate positive associations between fear-avoidance and pain (r = 0.01 to 0.65). Luque-Suarez et al. [49] included 50-cross-sectional studies in their review and reported very weak to moderate strength of association between kinesiophobia and pain in people with chronic musculoskeletal pain (r = 0.03 to 0.58). Taken together with findings of the current review, psychological factors and clinical outcomes appear to share a modest association for people with musculoskeletalal conditions. This suggests, that at least for some people with musculoskeletal conditions, psychological factors are associated with clinical outcome, and worthy of consideration. For example, some people may have low fear avoidance but high

pain ratings, and the reverse may also be true. Identifying people for whom psychological factors relate to pain, disability and functional outcomes may be important for a more targeted management approach.

In contrast to cross-sectional studies, longitudinal studies can evaluate whether psychological factors predict disease outcome over time. Studies of prediction require prospectively collected longitudinal data where the outcome is not present at enrolment [50]. This temporal sequence is important in determining cause and effect [51]. The data from the four longitudinal studies were inconsistent for the predictive value of psychological factors on long term clinical outcome (38, 42, 43, 45]. This may in part be explained by methodological heterogeneity including variability in tendon sites (4 sites), psychological factors measured (6 factors), clinical outcomes assessed (7 outcomes), and assessment periods (0 – 60 months).

It is also noteworthy that many people in the included trials had low baseline scores for the psychological constructs assessed. People with low baseline psychological scores may be less likely to display a relationship between their psychological status and clinical outcomes [52]. For example, baseline mean values for kinesiophobia (TSK) were reported as 23.7 points by Coombes et al. [43]; scores between 17-37 points are considered moderate risk of poor outcome [53, 54]. In the same study, participants recorded mean scores of 7.6 out of a total of 42 on the Hospital Anxiety and Depression Score (HADS), on which a score of 7 points (or lower) is considered not anxious or depressed [55]. Similarly, median baseline catastrophising was reported as 9 points by Kromer et al. [38], whereas scores >30 points are considered clinically relevant [56]. As higher levels of psychological factors at baseline may predict poorer clinical outcomes [54, 57], it appears this population may simply be underrepresented in the included studies; perhaps due to sampling bias or strict inclusion/exclusion criteria which often excludes participants with high levels of pain or bilateral pain symptoms [36, 38, 39].

Similarly to a prior review [21], we found substantial heterogeneity in relation to the range of psychological outcome measures across the included studies included in our review. For instance, depression and anxiety was measured in five studies, in which four different outcome measures were used. This lack of concensus restricts the synthesis and pooling of data for meta-analysis.

In accordance with other reviews [30, 48, 58, 59] there was consistency found in instruments used to measure fear avoidance (FABQ and kinesiophobia (TSK). Whilst the contributing role of fear avoidance behaviours has been well established in the development of chronic lower back pain and other musculoskeletal disorders [48, 60, 61], historically, this relationship has not been adequately examined in tendinopathy studies. Hence, it is possible that people with tendinopathy may not experience the same magnitude of fear and subsequent avoidance as do people with other musculoskeletal conditions, thus, these instruments may not adequately capture the lived experience of people with this musculoskeletal condition.

Strengths and weaknesses

Strengths of the review include pre-registering the protocol, a rigorous search strategy combined with using validated methods for evaluating risk of bias and the level of evidence. The review has been reported in accordance with PRISMA guidelines [23] and utilised two reviewers to screen and extract data. The main limitations relate to the literature. Most studies were cross-sectional with small samples, which are more prone to bias and confounding than prospective studies, and are unable to identify temporal relationships that may be causal. Second, high levels of heterogeneity prevented meta-analysis and limited comparisons between studies. Finally, detailed descriptive statistics were not always available, making the task of summarising these studies consistently challenging.

Future directions

First, there is a clear need for robust longitudinal studies to investigate the predictive value of psychological factors among people with tendinopathy. Second, significant heterogeneity of psychological outcome measures exist in selected studies resulting in inconsistency of reporting and thus preventing meta-analysis of the data; This lack of standardisation of outcome measures limits the usefulness of clinical trial evidence [62]. Within rheumatology, this problem has been addressed through the implementation of the Outcome Measures Rheumatology initiative (OMERACT), which has markedly improved outcome measurement for many rheumatologic conditions. This has been achieved by developing widely endorsed "core outcome measurement sets" (COMS) that are to be reported in all RCT and longitudinal studies [62, 63]. Recently, the International Scientific Tendinopathy Symposium Consensus (ICON) group have identified nine health related core domains, one being psychological factors [64]. Future studies are required to establish which psychological instruments would be applicable to use in the chosen setting. However, first we need to determine the specific psychological constructs that should be measured among people with tendinopathy and establish whether currently available instruments are suitable and fit for purpose. Finally, future validation studies are required to determine how many people have clinically meaningful psychological factors in tendinopathy and identify appropriate cutoff levels for currently used instruments. Proposed cut-off values have been established for the FABQ [65] and TSK [52] for LBP, however, none currently exist in tendinopathy. Identifying individuals who may be at low, moderate or high risk of poor outcome would allow clinicians to adapt their management strategies accordingly.

Conclusion

Synthesis of the cross-sectional data revealed very low certainty of evidence suggesting weak to moderate strength of associations between psychological factors and clinical outcomes in tendinopathy. Importantly, data derived from cross-sectional studies reported similar strengths of association to those found in other common musculoskeletal conditions, such as low back pain. Scarce and conflicting longitudinal data failed to show a predictive relationship between baseline psychological factors and long-term outcome. Hence, larger longitudinal cohort studies are required to investigate the predictive value of psychological factors upon long-term clinical outcome in tendinopathy populations, ensuring studies include people with meaningful levels of pain and psychological distress. Additionally, less heterogeneity of psychological outcome measures used in tendinopathy research is required; the development of a tendon specific measure may assist with this.

References

1. Scott A, Squier K, Alfredson H, Bahr R, Cook JL, Coombes, B, et al. Icon 2019: International Scientific Tendinopathy Symposium Consensus: Clinical Terminology. British Journal Of Sports Medicine**.** 2019**;** Bjsports-2019-100885.

2. De Jonge S, Van Den Berg, C, De Vos R, Van Der Heide H, Weir A, Verhaar J, Bierma-Zeinstra, S, Tol J. Incidence Of Midportion Achilles Tendinopathy In The General Population. Br J Sports Med. 2011;45**:**1026-1028.

3. Albers, I. S., Zwerver, J., Diercks, R. L., Dekker, J. H. & Van Den Akker-Scheek, I. Incidence and prevalence of lower extremity tendinopathy in a Dutch general practice population: a cross sectional study. BMC musculoskeletal disorders. 2016;17**:**16. doi:10.1186/s12891-016-0885-2

4. Shiri R, Viikari-Juntura E, Varonen H, Heliövaara M. Prevalence And Determinants Of Lateral And Medial Epicondylitis: A Population Study. American Journal Of Epidemiology. 2006;164**:**1065-1074.

5. Vos, T., Flaxman, A. D., Naghavi, M., Lozano, R., Michaud, C., Ezzati, M., Shibuya, K., Salomon, J. A., Abdalla, S. & Aboyans, V. Years Lived With Disability (Ylds) For 1160 Sequelae Of 289 Diseases And Injuries 1990–2010: A

Systematic Analysis For The Global Burden Of Disease Study 2010. The Lancet. 2012;380**:**2163-2196.

6. Littlewood C, May S, Walters S. 2013. Epidemiology Of Rotator Cuff Tendinopathy: A Systematic Review. Shoulder & Elbow. 2013:5**:**256-265.

7. Malliaras P, Barton CJ, Reeves ND, Langberg H. Achilles And Patellar Tendinopathy Loading Programmes. Sports Medicine. 2013:43**:**267-286.

8. Murphy M, Travers M, Gibson W, Chivers P, Debenham J, Docking S, Rio E. Rate Of Improvement Of Pain And Function In Mid-Portion Achilles Tendinopathy With Loading Protocols: A Systematic Review And Longitudinal Meta-Analysis. Sports Medicine. 2018;48**:**1875-1891.

9. Alfredson, H., Pietilä, T., Jonsson, P. & Lorentzon, R. 1998. Heavy-load eccentric calf muscle training for the treatment of chronic Achilles tendinosis. The American journal of sports medicine.1998;26:360-366.

10. Sayana MK, Maffulli, N. Eccentric calf muscle training in non-athletic patients with Achilles tendinopathy. Journal of Science and Medicine in Sport. 2007;10**:**52-58.

11. Sevier T, Stegink-Jansen C. Astym treatment vs eccentric exercise for lateral elbow tendinopathy: a randomized controlled trial. PeerJ. 2015;3:e967 https://doi.org/10.7717/peerj.967

12. Foster, N, Thomas, E, Bishop, A, Dunn K, & Main C. Distinctiveness of psychological obstacles to recovery in low back pain patients in primary care. Pain. 2010;148**:** 398-406.

13. Wierike S, van der Sluis A, van der Akker-Scheek A, Elferink-Gemser M, Visscher C. Psychological Factors Influencing the Recovery of Athletes with Anterior Cruciate Ligament Injury: A Systematic Review. Scandanavian Journal of Medicine & Science in Sports. 2012;23:527-540

14. Lin I, Wiles L, Waller R, Goucke R, Nagree Y, Gibberd, M, Straker L, Maher CG, O'Sullivan PP. What does best practice care for musculoskeletal pain look like? Eleven consistent recommendations from high-quality clinical practice guidelines: systematic review. British Journal of Sports Medicine. 2019:54**:**79-86.

15. Helminen EE, Arokoski JP, Selander TA, Sinikallio SH. Multiple psychological factors predict pain and disability among community-dwelling knee osteoarthritis patients: a five-year prospective study. Clin Rehabil. 2020;34(3):404-415. doi:10.1177/0269215519900533

16. McAuliffe S, Synott A, Casey H, Mc Creesh K, Purtill H, O'sullivan K. Beyond the tendon: Experiences and perceptions of people with persistent Achilles tendinopathy. Musculoskeletal Science & Practice. 2017;29**:**108-114.

17. Gillespie M, Macznik A, Wassinger C, Sole G. Rotator cuff related pain: Patient's understanding and experiences. Musculoskeletal Science and Practice. 2017;30:64-71

18. Turner J, Malliaras P, Goulis J, McAuliffe S. It's dissapointing and it's pretty frustrating because it feels like it's something that will never go away". A qualitative study exploring individual's beleifs and experiences of Achilles tendinopathy. PLoS ONE. 2020;15(5):e0233459.https:// doi.org/10.1371/journal.pone.0233459

19. Stephens G, O'Neill S, Mottershead C, Hawthorn C, Yeowell G, Littlewood C. "It's just like a needle going into my hip, basically all of the time". The experiences and perceptions of patients with Greater Trochanteric Pain Syndrome in the UK National Health Service. Musculoskeletal Science and Practice. 2020; 47, 1-7

20. Plinsinga ML, Coombes BK, Mellor R, Nicolson P, Grimaldi A, Hodges, P, Bennell, K, Vicenzino B. Psychological Factors Not Strength Deficits Are Associated With Severity Of Gluteal Tendinopathy: A Cross-Sectional Study. European Journal of Pain. 2018;22:1124-33

21. Mallows, A, Debenham, J, Walker T, Littlewood C. Association Of Psychological Variables And Outcome In Tendinopathy: A Systematic Review. British Journal Of Sports Medicine. 2017;51**:**743-748.

22. Drake C, Mallows A, Littlewood C. Psychosocial varables and presence, severity and prognosis of plantar heel pain: A systematic review of croess-sectional and prognostic associations. Musculoskeletal Care. 2018;16:329-338

23. Moher D, Liberati A, Telzlaff J, Altman D. The PRISMA Group. Preferred reporting items for systematic reviews and analysis: the PRIOSMA statement. PLOS Med. 2009;6(7): e1000097.doi:10.1371/jouurnal.pmed 1000097

24. Edwards R, Dworkin R, Sullivan M, Turk D, & Wasan A. The role of psychosocial processes in the development and maintenance of chronic pain. The Journal of Pain. 2016;17:T70-T92.

25. Turk, D. C., Fillingim, R. B., Ohrbach, R, Patel, K. V. Assessment of psychosocial and functional impact of chronic pain. The Journal of Pain. 2016;17:T21-T49.

26. Wells, G. A, Shea, B., O'Connel, D. Peterson J, Welch V, Losos M, et al. The Newcastle-Ottawa scale (NOS) for assessing the quailty of nonrandomised studies in meta-analyses.2009; http://www ohri ca/programs/clinical_epidemiology/oxford htm 2009 Feb

27. Zeng X, Zhang Y, Kwong JS, Zhang C, Li S, Sun F, et al. The methodological quality assessment tools for preclinical and clinical studies, systematic review and meta- analysis, and clinical practice guideline: a systematic review. Journal of evidence-based medicine. 2015;8:2-10

28. Bawor, M., Dennis, B. B., Anglin, R., Steiner, M., Thabane, L. & Samaan, Z. Sex differences in outcomes of methadone maintenance treatment for opioid addiction: a systematic review protocol. Systematic Reviews. 2014;3:E344 - 51.

29. Martinez-Calderon J, Meeus M, Struyf F, Morales-Asencio JM, Gijon-Nogueron G, Luque-Suarez A. The role of psychological factors in the perpetuation of pain intensity and disability in people with chronic shoulder pain: a systematic review. BMJ open. 2018;8:e020703.doi:10.1136/bmjopen-2017-020703

30. Martinez-Calderon J, Flores-Cortes M, Morales-Asencio JM, Luque-Suarez A. Pain-related fear, pain intensity and function in individuals with chronic musculoskeletal pain: a systematic review and meta-analysis. The Journal of Pain. 2019;20(12):1394-1415

31. Balshem, H., Helfand, M., Schünemann, H. J., Oxman, A. D., Kunz, R., Brozek, J., Vist, G. E., Falck-Ytter, Y., Meerpohl, J. & Norris, S. GRADE guidelines: 3. Rating the quality of evidence. Journal of clinical epidemiology. 2011;64: 401-406.

32. Guyatt G, Oxman A, Sultan S, Galsziou P, Akl E, Aloso-Coello P, Atkins D et al., GRADE guidelines: 9. Rating up the quality of evidence. Journal of Clinical Epidemiology 2011;64(12):1311-1316

33. Hinkle DE, Wiersma W, Jurs SG. Applied Statistics for the Behavioral Sciences 5th ed. Boston: Houghton Mifflin; 2003

34. R Core Team. R: A language and environment for statistical computing. R Foundation for Statistical Computing. 2018;Vienna, Austria. URL https://www.R-project.org/

35. Fletcher T. Psychometric: Applied Psychometic Theory.2015; R Package version 2.2

36. Engebretsen K, Grotle M, Bautz-Holter E, Ekeberg M, Brox J. Determinants Of The Shoulder Pain And Disability Index In Patients With Subacromial Shoulder Pain. Journal Of Rehabilitation Medicine. 2010;42:499-505.

37. Akyol, Y., Ulus, Y., Durmus, D., Tander, B., Cantu, R. F., Bilgici, A., Kuru, O. & Bek, Y. Shoulder Muscle Strength In Patients With Subacromial Impingement Syndrome: Its Relationship With Duration Of Quality Of Life And Emotional Status. Turkiye Fiziksel Tip Ve Rehabilitasyon Dergisi. 2013;59:176-181.

38. Kromer TO, Sieben JM, De Bie RA, Bastiaenen CH. Influence Of Fear-Avoidance Beliefs On Disability In Patients With Subacromial Shoulder Pain In Primary Care: A Secondary Analysis. Physical Therapy. 2014;94:1775-1784.

39. Maestroni L, Marelli, M, Gritti M, Civera F, Rabey M. Is Rotator Cuff Related Shoulder Pain A Multidimensional Disorder? An Exploratory Study. Scandinavian Journal Of Pain (published online ahead of print). 2019;20190108. doi: https://doi.org/10.1515/sjpain-2019-0108

40. Cotchett, M. P., Whittaker, G. & Erbas, B. Psychological Variables Associated With Foot Function And Foot Pain In Patients With Plantar Heel Pain. Clinical Rheumatology. 2015;34:957-964.

41. Cotchett, M., Lennecke, A., Medica, V. G., Whittaker, G. A. & Bonanno, D. R. The Association Between Pain Catastrophising And Kinesiophobia With Pain And Function In People With Plantar Heel Pain. The Foot. 2017;32:8-14.

42. Harutaichun, P, Pensri P, Boonyong S. Physical And Psychological Predictors On Pain Intensity In Conscripts With Plantar Fasciitis. Brazilian Journal Of Physical Therapy. 2019. https://doi.org/10.1016/j.bjpt.2019.02.014

43. Coombes, B. K., Bisset, L. & Vicenzino, B. Cold Hyperalgesia Associated With Poorer Prognosis In Lateral Epicondylalgia: A 1-Year Prognostic Study Of Physical And Psychological Factors. Clinical Journal Of Pain. 2015;31:30- 35.

44. Ferrer-Pena, R, Moreno-Lopez, M, Calvo-Lobo, C, Lopez-De-Uralde-Villanueva I, Fernandez-Carnero J. Relationship Of Dynamic Balance Impairment With Pain-Related And Psychosocial Measures In Primary Care Patients With Chronic

Greater Trochanteric Pain Syndrome. Pain Medicine (Malden, Mass.). 2019;20:810-817.

45. Silbernagel K, Brorsson A, Lundberg M. The majority of patients with Achilles tendinopathy recover fully when treated with exercise alone. The American Journal of Sports Medicine. 2010;39(3):607- 613.

46. Sterne JAC, Hernán MA, McAleenan A, Reeves BC, Higgins JPT. Chapter 25: Assessing risk of bias in a non-randomized study. In: Higgins JPT, Thomas J, Chandler J, Cumpston M, Li T, Page MJ, Welch VA (editors). Cochrane Handbook for Systematic Reviews of Interventions version 6.0 (updated July 2019). Cochrane, 2019. Available from www.training.cochrane.org/handbook.

47. Hill AB. The environment and disease: association or causation? Proc R Soc Med. 1965;58:295–300

48. Kroska E. 2016. A meta-analysis of fear avoidance and pain intensity: the paradox of chronic pain. Scandanavian Journal of Pain. 2016;13:43-58

49. Luque-Suarez A, Martinez-Calderon Javier, Falla D. Role of kinesiophobia on pain, disability and quality of life in people suffering from chronic musculoskeletal pain: a systematic review. Br.J.Sports.Med. 2019a;53:554-559

50. Kent P, Cancelliere C, Boyle E, Cassidy J, Kongsted A. A conceptual framework for prognostic research. BMC Medical Research Methodology. 2020; https://doi.org/10.1186/s12874-020-01050-7

51. Caruana EJ, Roman M, HernándezSánchez J, Solli P. Longitudinal studies. J Thorac Dis. 2015;7(11):E537-E540. doi: 10.3978/j.issn.2072-1439.2015.10.63

52. Beneciuk J, Hill J, Campbell P, et al. Identifying treatment effect modifiers in the STaRT Back Trial: a secondary analysis. J. Pain. 2017;18(1):54-65

53. Vlaeyen JWS, Kole-Snijders AMJ, Boeren RgB, van Eek H. Fear of movement/(re)injury in chronic low back pain and its relation to behavioral performance. Pain. 1995;62:363–372.

54. Wertli M, Rasmussen-Barr E, Weiser S, Bachmann L, Brunner F. Role of fear avoidance beliefs as a prognostic factor for outcome in patients with non-specific low back pain: a systematic review. The Spinal Journal. 2014;14:816-836

55. Bjelland I, Dahl AA, Haug TT, Neckelmann D. The validity of the Hospital Anxiety and Depression Scale. An updated literature review. J Psychosom Res. 2012;52:69–77

56. Sullivan M. The Pain Catastrophising Scale – user manual. Montreal, Quebeck: Sullivan, McGill University; 2009.

57. Picavet H, Vlaeyen J, Schouten J. Pain catastrophizing and kinesiophobia: predictors of chronic low back pain. American Journal of Epidemiology. 2002;156(11):1028-34
58. Zale, E, Lange K, Fields S, Ditre J. The relation between pain-related fear and disability: A meta-analysis. J Pain. 2013;14(10):1019-1030

59. Luque-Suarez A, Falla D, Morales-Asencio, Martinez-Calderon Javier. Is kinesiophobia and pain catastrophising at baseline associated with chronic pain and disability in whiplash-associated disorders? A systematic review. Br J Sports Med. 2019b; 0:1-7

60. Crombez G, Vlaeyen J, Heuts P, Lysens R. 1999. Pain-related fear is more disabling than pain iyself: evidence on the role of pain-related fear in chronic back pain disability. Pain. 1999;80:329-39

61. Cleleand J, Fritz J, Brennan G. Predictive validity of initial fear avoidance beliefs in patients with low back pain receiving physical therapy: is the FABQ a useful screening tool for identifying patients at risk for a poor recovery? Eur Spain J. 2008;17:70-79

62. Boers M, Kirwan J, Wells G, Beaton D, GossecL, d'Agostino M et al. Developing core outcome measurement sets for clinical trials: OMERACT Filter 2.0. Journal of Clinical Epidemiology. 2014;67:745-53

63. Tugwell P, Boers M, Brooks P, Simon L, Strand V, Idzerda L.OMERACT: an international initiative to improve outcome measurement in rheumatology. Trials. 2007;8:38

64. Vincenzino B, Jan-de Vos R, Alfredson H, Bahr R, Cook JL, BK Coombes, et al. ICON 2019 – International Scientific Tendinopathy Symposium Consensus: There are nine core health – related domains for tendinopathy (CORE DOMAINS): deplhi study of healthcare professionals and patients. Br J Sports Med. 2019;0:1-8.

65. George S, Fritz J, Childs J. Investigation of elevated fear-avoidance beliefs for patients with low back pain: a secondary analysis involving patients enrolled in physical therapy clinical trials. J Orthop Sports Phys Ther. 2008;38:50-8

Appendix 1

Database(s): Embase Classic+Embase 1947 to 2020 July 02
Search Strategy:

#	Searches	Results
1	exp tendinitis/	18220
2	tendinitis.tw.	3270
3	tend?nopath$.mp. [mp=title, abstract, heading word, drug trade name, original title, device manufacturer, drug manufacturer, device trade name, keyword, floating subheading word, candidate term word]	5813
4	exp achilles tendinitis/	1535
5	achilles tend?n$.tw.	11668
6	paratenonitis.mp. [mp=title, abstract, heading word, drug trade name, original title, device manufacturer, drug manufacturer, device trade name, keyword, floating subheading word, candidate term word]	82
7	peritend?n$.mp. [mp=title, abstract, heading word, drug trade name, original title, device manufacturer, drug manufacturer, device trade name, keyword, floating subheading word, candidate term word]	943
8	jumpers knee.mp. [mp=title, abstract, heading word, drug trade name, original title, device manufacturer, drug manufacturer, device trade name, keyword, floating subheading word, candidate term word]	327
9	exp epicondylitis/	4048
10	epicondyl$.tw.	5600
11	exp tennis elbow/	2966
12	tennis elbow.tw.	1282
13	lateral epicondyl$.mp. [mp=title, abstract, heading word, drug trade name, original title, device manufacturer, drug manufacturer, device trade name, keyword, floating subheading word, candidate term word]	2356
14	exp shoulder impingement syndrome/	2830
15	shoulder impingement syndrom$.tw.	430
16	exp rotator cuff injury/	11643
17	rotator cuff injur$.tw.	527
18	rotator cuff disease.mp. [mp=title, abstract, heading word, drug trade name, original title, device manufacturer, drug manufacturer, device trade name, keyword, floating subheading word, candidate term word]	602
19	greater trochanteric pain syndrom$.mp. [mp=title, abstract, heading word, drug trade name, original title, device manufacturer, drug manufacturer, device trade name, keyword, floating subheading word, candidate term word]	291
20	exp fear/	274006
21	fear$.tw.	114290
22	depression.tw.	474458
23	exp anxiety/	219742
24	anxiety.tw.	277800
25	exp catastrophizing/	3552

26	exp coping behavior/	64568
27	coping behavio$.tw.	2499
28	exp avoidance behavior/	36185
29	avoidance behavi$.tw.	5463
30	exp avoidance behavior/	36185
31	avoidance behavi$.tw.	5463
32	(fear adj1 avoidance).mp. [mp=title, abstract, heading word, drug trade name, original title, device manufacturer, drug manufacturer, device trade name, keyword, floating subheading word, candidate term word]	1787
33	(psycholog$ adj1 distress).mp. [mp=title, abstract, heading word, drug trade name, original title, device manufacturer, drug manufacturer, device trade name, keyword, floating subheading word, candidate term word]	25744
34	kinesiophobia.mp. [mp=title, abstract, heading word, drug trade name, original title, device manufacturer, drug manufacturer, device trade name, keyword, floating subheading word, candidate term word]	1258
35	hypervigilance.mp. [mp=title, abstract, heading word, drug trade name, original title, device manufacturer, drug manufacturer, device trade name, keyword, floating subheading word, candidate term word]	1063
36	catastrophi?ation.tw.	195
37	catastrophi?ing.mp. [mp=title, abstract, heading word, drug trade name, original title, device manufacturer, drug manufacturer, device trade name, keyword, floating subheading word, candidate term word]	5704
38	exp depression/	483960
39	exp emotion/	619158
40	emotion$.tw.	271747
41	psycholog$.mp. [mp=title, abstract, heading word, drug trade name, original title, device manufacturer, drug manufacturer, device trade name, keyword, floating subheading word, candidate term word]	1097787
42	tend?nosis.mp. [mp=title, abstract, heading word, drug trade name, original title, device manufacturer, drug manufacturer, device trade name, keyword, floating subheading word, candidate term word]	1396
43	subacromial pain.mp. [mp=title, abstract, heading word, drug trade name, original title, device manufacturer, drug manufacturer, device trade name, keyword, floating subheading word, candidate term word]	160
44	self efficacy.mp.	33977
45	exp plantar fasciitis/	1689
46	plantar heel pain.mp. [mp=title, abstract, heading word, drug trade name, original title, device manufacturer, drug manufacturer, device trade name, keyword, floating subheading word, candidate term word]	295
47	exp physiological stress/ or exp stress/	337643
48	1 or 2 or 3 or 4 or 5 or 6 or 7 or 8 or 9 or 10 or 11 or 12 or 13 or 14 or 15 or 16 or 17 or 18 or 19 or 42 or 43 or 45 or 46	48811
49	20 or 21 or 22 or 23 or 24 or 25 or 26 or 27 or 28 or 29 or 30 or 31 or 32 or 33 or 34 or 35 or 36 or 37 or 38 or 39 or 40 or 41 or 44 or 47	2348181
50	48 and 49	1380

Figure 1
Click here to access/download;Figure;Figure 1 PRISMA 2009 flow diagram.tiff

Fig 1. PRISMA Flow Diagram

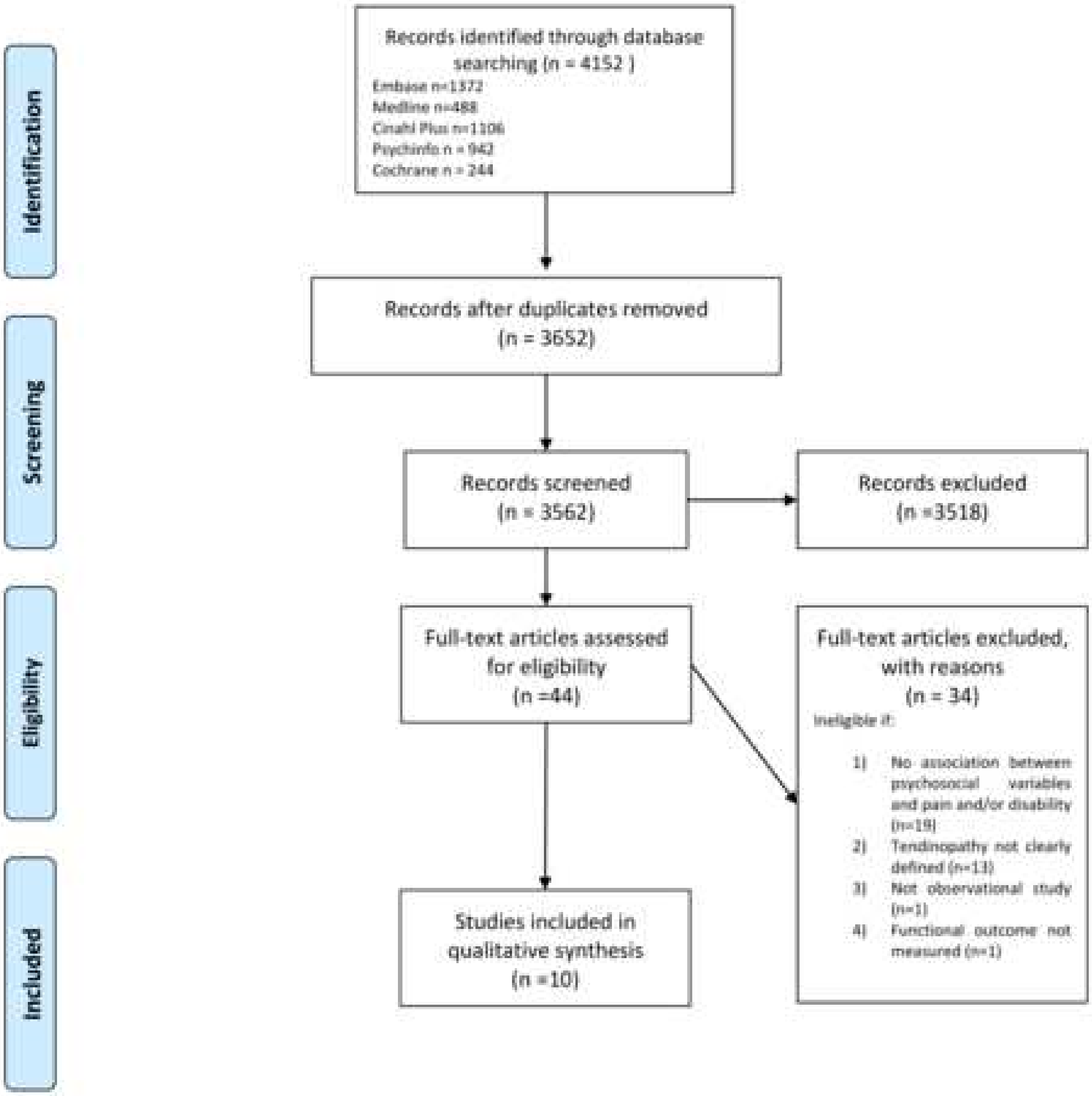

Moher D, Liberati A, Tetzlaff J, Altman DG, The PRISMA Group (2009). *Preferred Reporting Items for Systematic Reviews and Analyses*: The PRISMA Statement. PLoS Med 6(7): e1000097. doi:10.1371/journal.pmed1000097

For more information, visit www.prisma-statement.org.

PRISMA Checklist

Click here to access/download
Supporting Information
PRISMA 2009 checklist.doc

Click here to access/download
Supporting Information
Financial Disclosure Statement (1).docx
