# Supplementary material for: The strength of association between psychological factors and clinical outcome in tendinopathy: A systematic review
Source: PLoS One. 2020 Nov 30;15(11):e0242568. doi: 10.1371/journal.pone.0242568 (PMC7703922; doi:10.1371/journal.pone.0242568)
Supplement: Attachment — Submitted filename: Paper review PlosOne PONE-D-20-23274.docx [file pone.0242568.s003.docx]

**Paper review: Manuscript Number PONE-D-20-23274**

**The strength of association between psychological factors and clinical outcome in tendinopathy: a systematic review**

Thanks for giving me the opportunity to review this paper.

This is a very interesting systematic review paper reporting the magnitude of the association between psychological factors and clinical outcome in tendinopathy. The review identified ten studies that were assessed for quality using the modified Newcastle Ottawa Scale and certainty evidence by the GRADE Criteria. However there are some areas that the authors need to correct and adjust for more clarity as described below.

**Title and abstract**

1. The title is well written and captures all the necessary information from the study.
2. The abstract provides an informative and balanced summary of the appraisal. There is a need to provide the implication of the review for current and future practice.
3. It might not be requirement of the journal, but most of the time the registration number is provided at the end of the abstract.

**Introduction**

4. The introduction is well written explaining the rationale of the study.
5. It could be good to mention the setting where the study by Albers et al. was conducted (line 56-58). The same for the other studies in the same line.
6. I may suggest to mention the other treatment modalities used though exercises might have been shown to be effective in the management of tendinopathy (Line 59-66).
7. There is a need to elaborate more the problems related to the association focusing at methodological variability of the studies to support the idea of investigating the “strength of the association” (Line 76-78)
8. Review sentence (Line 76-78)

**Methods and materials**

9. The methods section is well written with enough details.
10. There is a need to mention what was based on to categorize the time of the outcome.

**Results**

11. The results are well presented with enough details
12. Study selection: Did not mention whether there are records identified through other sources as mentioned in the method section (Same observation for Figure 1)
13. The values presented in the results section should always match the findings from the reviewed studies.
    a. Page 316: r=0.25 in text while in table it is r=0.27
    b. Page 325: β=-0.41 in text while in table it is *B* 0.41
    c. Page 344: r = -0.590 in text while in table it is r = ---590
14. Depending of where table 2 will be placed, the superscript reference number might change.

**Discussion**

15. The discussion summarises the main findings in the review and the strength of evidence for each main outcome
16. The example of musculoskeletal conditions used in the discussion is only low back pain and some other place rheumatological conditions. Can this mean that no other studies available that investigated the relationship between psychological factors and musculoskeletal pain or function.
17. What is the implication of the findings for practice recommendation?

**Other comments**

18. Line 9: “& These authors also contributed equally to this work” need review as there is only one author with “&” and it is better to mention the extent of the contribution.
19. Need consistency in referencing (check line 62, 73)
20. Corrections needed (Line 64)
21. Line 64: MSK should be written in full if first time used.
22. Insert reference (line 78-81)
23. Page 276: change “9-separate” into (nine separate)
24. Corrections needed (line 245, 317, 344, 424)
25. Line 353: “certatinty” into “certainty”
26. Line 359: “propsective” into “prospective”
27. Line 361: replace “our” with “the”
28. Line 363: “weak-to-moderate” into “weak to moderate”
29. Line 365: “50-cross-sectional” into “50 cross sectional”
