# Supplementary material for: The strength of association between psychological factors and clinical outcome in tendinopathy: A systematic review
Source: PLoS One. 2020 Nov 30;15(11):e0242568. doi: 10.1371/journal.pone.0242568 (PMC7703922; doi:10.1371/journal.pone.0242568)
Supplement: Attachment — Submitted filename: Response to Reviewers_SMA (2).docx [file pone.0242568.s004.docx]

Cover letter for revisions

**The strength of association between psychological factors and clinical outcome**

**in tendinopathy: a systematic review**

We thank the reviewers for their thoughtful and thorough review of our manuscript. We have taken every comment into consideration and responded to them individually below.

Note: Reviewer comments are in black text and author response and revisions in red. We have also provided a marked up copy of our manuscript that highlights all the changes made to the original version. This manuscript is titled ‘Revised Manuscript with Track Changes’.

**Reviewer 1**

Comment #1:

Abstract- insert 'factors' after psychological. line 26

Response #1

Thank you, we have now added ‘factors’ after psychological – line 26

Comment #2

Introduction- 'MSK'. First mention. Write in full and then abbreviate (Line 64) and since you have abbreviated be consistent using it thereafter.

Response #2

Thank you, the abbreviated MSK has now been written in full when it first appears as per request – line 56

Comment #3

Methods- (SPADI, GROC). write full names. line 123

Response #3

Thank you, full abbreviated names are written for SPADI and GROC – see lines 135-137

Comment #4

Table 2- Format the column heading of table 2. indicate what represent SD and unit. e.g. Mean age ±SD (year) and BMI± SD (unit). Delete SD in the rows.

Response #4

Thank you, formatting has been amended as requested in table 2. Deleted SD rows – see line 185

Comment #5

References- check the journal for list of reference specifications. Punctuation and use of et al. for authors more than six were not consistent.

Response #5

Thank you, reference check conducted and amended, and punctuation amended where needed.

**Reviewer #2**

Comment #1

Provide detailed search terms used in at least one database with how search terms were modified for other databases. Search terms used are limited to a few types of tendinopathies and body regions. They do not reflect the intention of review to include all types of tendinopathies for all body regions. Similarly, the search terms do not reflect all psychological factors.

Response #1

Thank you for your comment. It was not our intention to go through an exhaustive list of all specific terms for tendinopathy conditions, however It was our intention that the scope of the review included all the major search base terms for all reported psychological and tendinopathy conditions of interest. We relied upon the MESH base generic terms used to capture tendinopathies, such as, ‘tendinitis, tend?nopath$, tend?n$’ etc. Using these terms, we would have expected to capture the majority of conditions which have ‘tendon’ in their diagnosis e.g. ‘patella tendinopathy, Achilles tendinopathy, tibialis posterior tendinopathy, posterior tibial tendon dysfunction, etc’. In addition, we supplemented our database search with more commmon specific and ‘pseudo’ terms in order to capture other tendinopathy conditions which may not have been captured using base generic terms. Examples of these terms include ‘plantar heel pain, shoulder impingement syndrome, jumpers’ knee, tennis elbow, etc’. Overall, we feel that we were able to capture all tendinopathies of interest in our search.

We agree the term “psychological” is a broad term. The choice of Psychological keywords utilised in the current review were informed by existing literature in MSK pain – primarily low back pain given the huge body of literature in this area. In addition, we also referred to existing qualitive literature in tendinopathy to help identify potential psychological domains and keywords.

Finally we also considered and were guided by well cited and popular publications by Edwards et al 2016, Turk et al 2016 and Linton & Shaw 2011 regarding categorisation of psychological factors into cognitive, emotional and behavioural processes. Similar to the tendinopathy search, various psychological broad base generic terms were supplemented with more specific terms in order to capture the most commonly studied factors found in MSK conditions. Overall, we feel that we were able to capture all psychological factors of interest in our search.

As a result of the reviewer’s response we have noticed an oversight in which behavioural factors were not named in the psychological outcomes-for which we apologise. Consequently, behavioural factors have been added to the psychological factors in the methods section – please refer to line 122-123.

***“Behavioural factors*** *including but not limited to avoidance, maladaptive (negative coping -styles*”

Comment #2
Data synthesis: Did the authors use any framework or guidelines to perform Quality synthesis? This is a factor that might have contributed to the Results section that is slightly tedious to read because of a lack of a priori framework for qualitative evidence synthesis

Response #2

Thank you for your comment. In an effort to formalise the quality of the body of evidence of the studies selected, we utilised the GRADE criteria as recommended by the Cochrane handbook. Our GRADE criteria include a quality synthesis which is tabulated (Table 4) and detailed within the manuscript - line 228-255. I can also refer the reviewer to the Cochrane Handbook Part 8.2.2 in which Cochrane draws a distinction between quality and risk of bias assessment and encourages the focus on risk of bias and this is what we have done with the application of the GRADE.

In accordance with your suggestion regarding the results section, we attempted to minimise the content within the results section – see line 314. In addition, we also formulated a separate results table containing the documented correlation coefficients to assess if this would add clarity for the reader. However, on reflection, we felt that the addition of a separate results table added little benefit to the reader as it interrupted the flow, clarity and consistency of the text. We acknowledge that there are numerous ways of presenting result findings in systematic reviews, however, presenting them in this format is not uncommon (e.g. Luque-Suarez et al. 2019). We feel as we are investigating the strength of association between psychological factors and clinical outcome, we chose to represent the relevant data in a factual manner and reserve the interpretation of the results to the discussion section.

Comment #3

It is unclear how was heterogeneity defined, and why meta-analysis was not performed. A priori criteria that authors used as criteria for meta-analysis are necessary.

Response #3

Thank you for your comment. Heterogeneity between studies was identified as a limitation of our systematic review – see line 444-446. Synthesis of our data using meta- analysis was to be conducted, on the proviso that the included studies provided homogenous data – which were documented in PROSPERO (section 28). Unfortunately, meta-analysis was unable to be conducted in accordance with recommendations from Cochrane Handbook – due to diversity of outcomes and populations within the studies included in our review (Part 9.1.4). Areas of heterogeneity and the specific reasons why meta-analysis was not performed was highlighted in our review under section ‘Data Synthesis’ – Line 257-259. Methodological heterogeneity was also discussed in – Lines 406-409. Other references to identified heterogeneity of psychological outcome measures between studies was also highlighted - lines 424-428.

Comment #4

Risk of Bias and overall GRADE rating: It is surprising to read that 9 of 10 studies had low risk of bias, however, based on GRADE assessment each category was classified as "very low levels of certainty" especially attributing to the risk of bias (80%). Am I missing something here?

Response #4

Thank you for your comment. In reference to your comments we have noted an error within the reporting of the level of certainty with the GRADE. Rotator cuff related shoulder pain and lateral elbow tendinopathy were incorrectly reported as ‘very low certainty of evidence’ when in fact the correct level should be reported as ‘low certainty of evidence’. In view of this we have amended all text referring to the former and correctly re-evaluated the level of certainty to the latter – please refer to lines 38, 49, 228 (Table 4), 313, 323, 328, 352, 375, 378, 471.

In response to concerns raised regarding classification of the GRADE, due to limitations (lack of randomisation-suggesting high likelihood of bias) of our observational studies included in our review, all studies were assigned an a priori ranking of low quality of evidence as per the Grades recommendations (Cochrane Handbook 12.2.1 & 12.2.2). Hence, all our eligible studies which were of observational designs, started with a ‘low quality’ rating. Factors that influence rating up the quality of evidence are detailed in lines 251 – 255 and in Guyett et al (2011).

Minor Comments

Comment Abstract #1

Unclear what clinical outcomes were.

Response Abstract #1

Thank you for your comment. We have added addressed this concern by adding ‘pain and disability’ to the abstract. Please refer to line – 33

Comment Abstract #2

Stating how many authors performed screening, data extraction, and risk of bias assessment can be a good way to reflect the quality of the review through its Abstract.

Response to abstract #2

Thank you for your comment. We have attempted to address this point in the abstract – please refer to alteration on line 34

Comment abstract #3

Conclusions: new results should not appear in Conclusions: see reports on comparison with other musculoskeletal disorders and GRADE recommendations

Response to abstract #3

Thank you for your comment. We have altered the text in the conclusion section of the abstract as recommended by the reviewer.

Comment methods #1

Line 114 – I do not think that outcomes can be divided into Primary and Secondary for this type of review. These categories are better suited for reviews of RCTs.

Response methods #1

Thank you for your comment. We have adjusted the headings to read ‘psychological outcomes’ and ‘clinical outcomes’ please refer to line 114 & 125

Comments methods #2
SPADI, GROC - add full forms.

Response to methods #2

Thank you for your comment. Full forms of the SPADI and GROC have been included as recommended– please refer to lines 135-137

Comment to methods #3

GROC is not a measure of disability.

Response to methods #3

Thank you for your comment. We agree with the reviewer we have amended the text accordingly – please refer to line 137

Comment to methods #4

Timing of outcome measures: Are there any reasons how the authors chose to categorise timing of outcomes into short, medium and long term? These are not consistent with Cochrane reviews. Please provide appropriate citations. Clarify if these were set a priori.

Response to methods #4

Thank you for your comment. We are aware that categorising of outcome assessments is very context specific – and what is deemed short, medium and long-term timings can appear to be very arbitrary. We applied our timing of outcome assessment, a priori, – as these were the time frames that reflected previous studies in this area (Malliaras et al. 2020, Naunton et al. 2020).

We have amended our text accordingly and changed the terminology – citing relevant articles using the same time frames in similar reviews with similar clinical outcomes - please refer to line 147. To remove potential ambiguity, we have removed the time frames (short, medium and long term) from the text, as we are aware there is little consensus on what is regarded as short or long term, so the focus is on labelling the time period in our review in weeks and months, please refer to lines 148-150

*‘Psychological and clinical outcomes were extracted and categorised into timelines. The following timelines were established, a priori, based upon previous literature [27,28]’*

Comments methods #5

Line 148: unsure what the authors mean when they say ONE author "independently" screened the title and abstract. Independent of whom?

Response methods #5

Thank you for your comment. We have altered the manuscript and removed the word ‘independently’ – please refer to line 165

Comment methods #6

Line 160. It should be study design, not "type of trial" as observational studies were included.

Response methods #6

Thank you for your comment. We have amended the manuscript and removed the words ‘type of trial’ and replaced with ‘study design’, please refer to line 177

Comment #7

Cite the papers included in Table 4 that informs the GRADE recommendations.

Response methods #7

Thank you for your comment. Papers have been cited as requested and manuscript amended accordingly – please refer to Table 4.

Comments results #1

Overall, the results could read better if the authors follow a qualitative synthesis guideline or a framework.

Response results #1

Thank you for your comment. Please refer to ‘Reviewer 2 - Response #2’

Comment results #2
Lines 268-271- name what outcome measures were included.

Response results #2

Thank you for your comment. The original text has been updated and outcome measures have been included – please refer to lines 287-293.

*‘(5 outcome measures,* *Beck Depression Inventory, Hospital Anxiety and Depression Scale, Depression Anxiety Stress Scales, Hopkins Symptom Checklist-25, Outcome Evaluation Questionnaire) and cognitive (7 outcome measures,* *Tampa Scale of Kinesiophobia-17, Tampa Scale of Kinesiophobia -11, Tampa Scale of kinesiophobia Swedish Version -17, Patient Catastrophising Scale, Chronic Pain Self-Efficacy Scale, Fear Avoidance Belief Questionnaire, Patient and Impairment Relationship Scale)’*

In addition, we realised that an error had occurred in the text under the heading ‘outcome measures’ line 286. We had inadvertently miscalculated the amount of outcome measures used to measure psychological factors. We apologise for this error and it has been corrected – please refer to lines 286-294

Comment conclusion #1

Name what the clinical outcomes were to be unambiguous and explicit.

Response conclusion #1

Thank you for your comment. The original text has been updated to include ‘pain, disability and physical functional outcome’ in accordance with the reviewer’s request, please refer to lines 472-473

**Reviewer #3**

Comment #1

A minor suggestion is to explain the abbreviation MSK in the introduction.

Response #1

Thank you for your comment – we have altered the manuscript in accordance with your comment. Please refer to line 56

**Reviewer #4**

Comment Introduction #5

It could be good to mention the setting where the study by Albers et al. was conducted (line 56-58). The same for the other studies in the same line.

Response introduction #5

Thank you for your comment. We have altered the manuscript and included setting of study in text – please refer to line 59

Comment introduction #6

I may suggest to mention the other treatment modalities used though exercises might have been shown to be effective in the management of tendinopathy (Line 59-66).

Response introduction #6

Thank you for your comment. We have altered our manuscript to include heavy-slow resistance as another potential exercise alternative – we hope this addresses the concern - please refer to line 63.

Comment introduction #7

There is a need to elaborate more the problems related to the association focusing at methodological variability of the studies to support the idea of investigating the “strength of the association” (Line 76-78).

Response introduction #7

Thank you for your comment. In response to your comment we have altered the manuscript accordingly to further clarify our rationale for investigating the strength of association. Please refer to lines 80-87

*‘Several factors have been highlighted as potential explanations for the conflicting evidence base, such as, variance in population, heterogeneity of outcome measures and differing cognitive factors [21, 22]. Despite the ambiguity, previous reviews suggest specific psychological variables may be associated with tendinopathy and suboptimal outcomes. Interestingly, no data has yet been provided* *regarding the strength of association of psychological factors and outcome in people with tendinopathy and how they compare with other common MSK conditions, such as chronic low back pain’*

Comment Methods #10

There is a need to mention what was based on to categorize the time of the outcome.

Response introduction #10

Thank you for your comment. Please refer to Reviewer #2 - Response methods #4.

Comment results #12

Study selection: Did not mention whether there are records identified through other sources as mentioned in the method section (Same observation for Figure 1)

Response results #12

Thank you for your comment. We have updated the Prisma diagram to show records identified by other sources. We have also updated the Results section in the manuscript to clarify additional sources – please refer to line 265-266

Comment results #13

The values presented in the results section should always match the findings from the reviewed studies.
a. Page 316: r=0.25 in text while in table it is r=0.27
b. Page 325: β=-0.41 in text while in table it is B 0.41
c. Page 344: r = -0.590 in text while in table it is r = ---590

Response results #13

Thank you for your comment. All values have been updated in the manuscript. Please refer to lines, 341, 350 and in Table 2.

Comment Discussion #16

The example of musculoskeletal conditions used in the discussion is only low back pain and some other place rheumatological conditions. Can this mean that no other studies available that investigated the relationship between psychological factors and musculoskeletal pain or function.

Response Discussion #16

Thank you for the comment. We agree, the majority of studies investigating the role of psychological factors in chronic MSK conditions have been primarily conducted in the low back pain literature. The studies cited in our discussion included literature from a variety of MSK conditions. For example, Martinez-Calderon [30] and Luque-Suraz [52, 62] reviews, included studies investigating conditions such as chronic whiplash associated disorders, osteoarthritis of the knee, cervical pain, upper limb pain and chronic knee pain. We have altered the text in the manuscript to highlight the variances in chronic pain conditions. Please refer to line 392.

Comment discussion #17

What is the implication of the findings for practice recommendation?

Response discussion #17

Thank you for your comment. We did not include implications of the findings for practice recommendation. However, recommendations made in our review referred specifically to future research design and how we can reduce heterogeneity of the data in future tendinopathy studies. Furthermore, we also questioned whether the clinometric properties of instruments currently used to measure psychological factors are fit for purpose. Our findings and recommendations will hopefully create further questions which can be investigated in the future.

Comment ‘other comments’ #18

“& These authors also contributed equally to this work” need review as there is only one author with “&” and it is better to mention the extent of the contribution.

Response ‘other comments’#18

Thank you for your comment – we have added the ‘&’ as per PLoS One instructions.

Comment ‘other comments’ #19

Need consistency in referencing (check line 62, 73)

Response ‘other comments’#19

Thank you for your comment. We have altered text – please refer to line 65

Comment ‘other comments’ #20 & 21

Corrections needed (Line 64) & Line 64: MSK should be written in full if first time used.

Response ‘other comments’#20 & 21

Thank you for your comments. Musculoskeletal has been written in full, please refer to line 56.

Comments ‘other comments’ #22

Insert reference (line 78-81)

Response ‘other comments’ #22

Thank you for your comment. Text line 88-91 we have included a reference as suggested.

Comments ‘other comments’ #23 - 29

23. Page 276: change “9-separate” into (nine separate)
24. Corrections needed (line 245, 317, 344, 424)
25. Line 353: “certatinty” into “certainty”
26. Line 359: “propsective” into “prospective”
27. Line 361: replace “our” with “the”
28. Line 363: “weak-to-moderate” into “weak to moderate”
29. Line 365: “50-cross-sectional” into “50 cross sectional”

Response ‘other comments’ #22-29

Thank you for your comments – all amendments have been made.
